# Pharmacological Levels of Withaferin A (*Withania somnifera*) Trigger Clinically Relevant Anticancer Effects Specific to Triple Negative Breast Cancer Cells

**DOI:** 10.1371/journal.pone.0087850

**Published:** 2014-02-03

**Authors:** Katarzyna Szarc vel Szic, Ken Op de Beeck, Dariusz Ratman, An Wouters, Ilse M. Beck, Ken Declerck, Karen Heyninck, Erik Fransen, Marc Bracke, Karolien De Bosscher, Filip Lardon, Guy Van Camp, Wim Vanden Berghe

**Affiliations:** 1 Laboratory of Protein Chemistry, Proteomics and Epigenetic Signaling (PPES), Department of Biomedical Sciences, University of Antwerp, Antwerp, Belgium; 2 Center of Medical Genetics, Department of Biomedical Sciences, University of Antwerp, Antwerp, Belgium; 3 Center for Oncological Research (CORE), Laboratory of Cancer Research and Clinical Oncology, Department of Oncology, University of Antwerp, Antwerp, Belgium; 4 Nuclear Receptor Signaling Unit, Cytokine Receptor Laboratory, VIB Department of Medical Protein Research, Ghent University, Ghent, Belgium; 5 Laboratory of Experimental Cancer Research (LECR), Department of Radiation Therapy and Experimental Cancer Research, Ghent University, Ghent, Belgium; 6 Laboratory of Eukaryotic Gene Expression and Signal Transduction (LEGEST), Ghent University, Ghent, Belgium; 7 StatUa Center for Statistics, University of Antwerp, Antwerp, Belgium; University of Alabama at Birmingham, United States of America

## Abstract

Withaferin A (WA) isolated from *Withania somnifera* (Ashwagandha) has recently become an attractive phytochemical under investigation in various preclinical studies for treatment of different cancer types. In the present study, a comparative pathway-based transcriptome analysis was applied in epithelial-like MCF-7 and triple negative mesenchymal MDA-MB-231 breast cancer cells exposed to different concentrations of WA which can be detected systemically in *in vivo* experiments. Whereas WA treatment demonstrated attenuation of multiple cancer hallmarks, the withanolide analogue Withanone (WN) did not exert any of the described effects at comparable concentrations. Pathway enrichment analysis revealed that WA targets specific cancer processes related to cell death, cell cycle and proliferation, which could be functionally validated by flow cytometry and real-time cell proliferation assays. WA also strongly decreased MDA-MB-231 invasion as determined by single-cell collagen invasion assay. This was further supported by decreased gene expression of extracellular matrix-degrading proteases (uPA, PLAT, ADAM8), cell adhesion molecules (integrins, laminins), pro-inflammatory mediators of the metastasis-promoting tumor microenvironment (TNFSF12, IL6, ANGPTL2, CSF1R) and concomitant increased expression of the validated breast cancer metastasis suppressor gene (*BRMS1*). In line with the transcriptional changes, nanomolar concentrations of WA significantly decreased protein levels and corresponding activity of uPA in MDA-MB-231 cell supernatant, further supporting its anti-metastatic properties. Finally, hierarchical clustering analysis of 84 chromatin writer-reader-eraser enzymes revealed that WA treatment of invasive mesenchymal MDA-MB-231 cells reprogrammed their transcription levels more similarly towards the pattern observed in non-invasive MCF-7 cells. In conclusion, taking into account that sub-cytotoxic concentrations of WA target multiple metastatic effectors in therapy-resistant triple negative breast cancer, WA-based therapeutic strategies targeting the uPA pathway hold promise for further (pre)clinical development to defeat aggressive metastatic breast cancer.

## Introduction

Despite the tremendous advances made by screening programs as well as a slight decrease in incidence in the last decade, breast cancer remains the most frequently diagnosed cancer among women in Western societies and the second leading cause of cancer-related deaths worldwide [1]. Remarkably, the vast majority of diagnosed breast cancers (85%) are neither linked to genetic mutation of *BRCA1* or *BRCA2* genes nor to a family history of such malignancy. Notwithstanding the existence of several breast cancer chemotherapeutics, such as doxorubicine, paclitaxel, or selective estrogen modulators (e.g. tamoxifen or raloxifene), the latter remain highly ineffective in treating triple negative breast cancers (TNBC), which are devoid of estrogen receptor, progesterone receptor and human epidermal growth factor receptor 2 (HER2/neu). These cancers form a heterogeneous group of the most invasive cancers and remain the main obstacle in breast cancer treatment [2], [3]. Therefore, clinical development of multifunctional therapeutics that would block the growth and metastasis of transformed breast cells irrespective of their receptor status, and that would be less cytotoxic to healthy, surrounding cells than standard chemotherapeutics, is of great interest. Some plant compounds and their secondary metabolites fulfill the abovementioned criteria. They exhibit strong anti-inflammatory and anticancer effects while showing minor side effects, especially during long-term exposure.

Withaferin A (WA), the main constituent of *Withania somnifera* Dunal (also called Ashwagandha or Indian winter cherry), belongs to the class of steroidal lactone metabolites (withanolides), which play an important role in plant responses to pathogens, drought or low temperature [4]. Various mechanisms have been proposed to explain the anti-tumor activity of WA, including potent anti-inflammatory, anti-angiogenic, anti-metastatic, pro-apoptotic and radiosensitizing properties (reviewed in [5], [6]). With respect to breast cancer, WA and extracts of *Withania somnifera* were reported to inhibit the viability and growth of several breast cancer cell lines including ER-positive T-47D, MCF-7, MCF-7/BUS cells, and triple negative MDA-MB-231, Sk-Br-3 cells [7] as well as MDA-MB-231 human breast cancer xenografts *in vivo* [8]. Moreover, recently WA's efficacy in a mammary tumor virus-neu transgenic mouse model was studied [9]. The underlying mechanism responsible for anticancer properties of WA is not completely understood, but recent studies have demonstrated that WA causes G2/M cell cycle arrest or apoptosis via a FOXO3a- and BIM- dependent mechanism, via inhibition of Hsp90 and via induction of ROS production [8], [10]–[12]. In metastatic breast cancer models, WA has been shown to promote disassembly of vimentin, an intermediate filament protein [13]. However, the specificity of this interaction has recently been contested. Hence, effects of WA on other intermediate filaments could also play a role [14]. Moreover, Lee et al. have currently proposed that decreased breast cancer cell migration following WA treatment might result from Notch pathway activation [15]. Finally, we, and others, have shown that WA inhibits constitutive and inducible activity of NF-*κ*B and FOSL1 (Fra1) in MDA-MB-231 cells [16], [17]. These genes, together with STAT3 and IL-6, mediate chronic inflammatory responses in the tumor microenvironment, thus promoting survival of malignant cells [18]. Since most published studies focus on targeting breast cancer cell lines in general, irrespective of their receptor expression status, the present study was undertaken to identify key biological processes and novel molecular target genes affected by WA in triple negative metastatic breast cancer cells (MDA-MB-231) as compared to non-invasive ER-positive MCF-7 cells. Moreover, to demonstrate WA's specific effects, we investigated the anticancer activity of another closely related withanolide, Withanone (WN). In order to find unique cell line-specific gene expression profiles we applied an integrated systems biology approach by whole genome gene expression profiling (Human HT-12 v4 BeadChip, Illumina) and subsequent Ingenuity Pathway network analysis (IPA).

## Results

### WA decreases cell viability of cultured breast cancer cells in a time- and concentration- dependent manner

**Figure 1:**
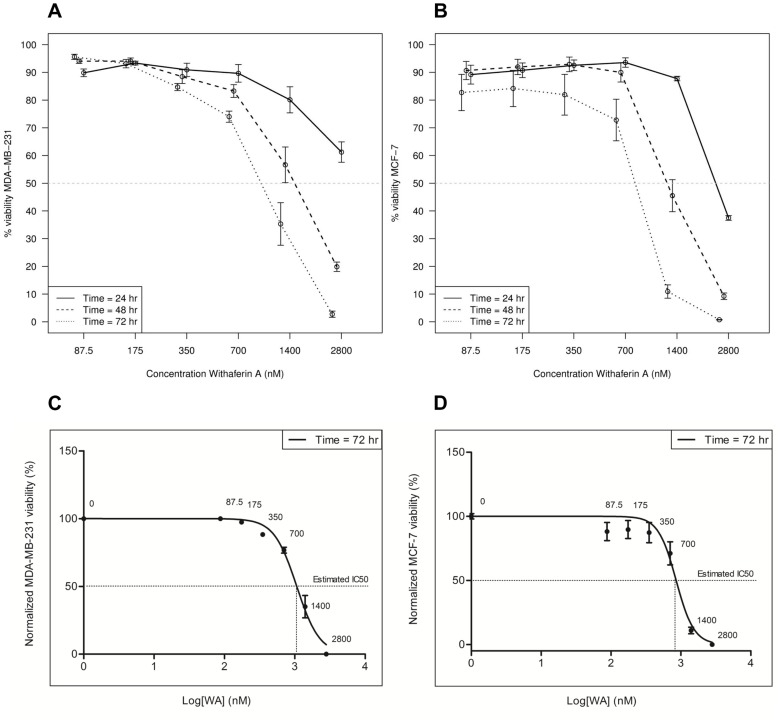
Effect of WA treatment on MDA-MB-231 and MCF-7 cell viability. **(A**–**B)** Dose-response curves of GelRed™ positive/negative MDA-MB-231 and MCF-7 cells treated every 24 hours with increasing concentrations of WA for a total time of 24, 48 and 72 hours as determined by FACS analysis. Data represent (mean ± SEM) values of three independent experiments. Observed IC_50_ values for each time point are marked with a dashed line. **(C**–**D)** Estimation of IC_50_ values at 72 hours exposure time was performed in GraphPad Prism version 5.00 for Windows using dose-response curve fitting (log(inhibition) vs normalized response (variable slope)) of MDA-MB-231 and MCF-7 cells treated every 24 hours with different doses of WA for a total time of 72 hours. Observations were normalized to a DMSO control, here represented as 100% viability.

In a first series of experiments, we assessed global cellular cytotoxicity of WA at nM to μM concentrations in MCF-7 and MDA-MB-231 cells. Cell death in breast cancer cells exposed to different concentrations of WA or solvent for 24, 48 or 72 h was determined by Gel Red staining and subsequent FACS analysis. A time- and concentration-dependent decrease in cell viability was revealed and expressed as decrease in Gel Red negative cell fraction as shown in **Figure 1A–B**. To estimate the IC_50_ concentration at 72 hours we normalized cell viability from 100% in solvent control to 0% in the highest concentration of WA. For 24 h and 48 h exposure, on the other hand, considerable viability remained even at the highest WA concentrations. Therefore IC_50_ values were not formally estimated. MCF-7 cells revealed the highest sensitivity to WA treatment and an IC_50_ value of 853.6 nM was estimated, with 95% CI ranging from 722.8 nM to 1008.0 nM. MDA-MB-231 cells showed to be less sensitive to WA treatment and an IC_50_ value of 1066 nM was estimated, with 95% CI ranging from 976.2 nM to 1164.0 nM **(** **Figure 1C**–**D** **)**. For further molecular studies and gene expression profiling in the breast cancer cells, a sub-cytotoxic concentration of 700 nM was selected, which is therapeutically relevant and effective in cancer xenograft studies *in vivo* [13]. Furthermore, as low concentrations of natural anticancer compounds can elicit stable changes in gene expression through epigenetic mechanisms which are transmitted through the cell cycle [19], WA was administered repeatedly, every 24 h, to expose various cell generations.

### Hierarchical clustering analysis of WA-responsive genes identifies a highly reproducible set of breast cancer-specific anticancer responses

**Figure 2:**
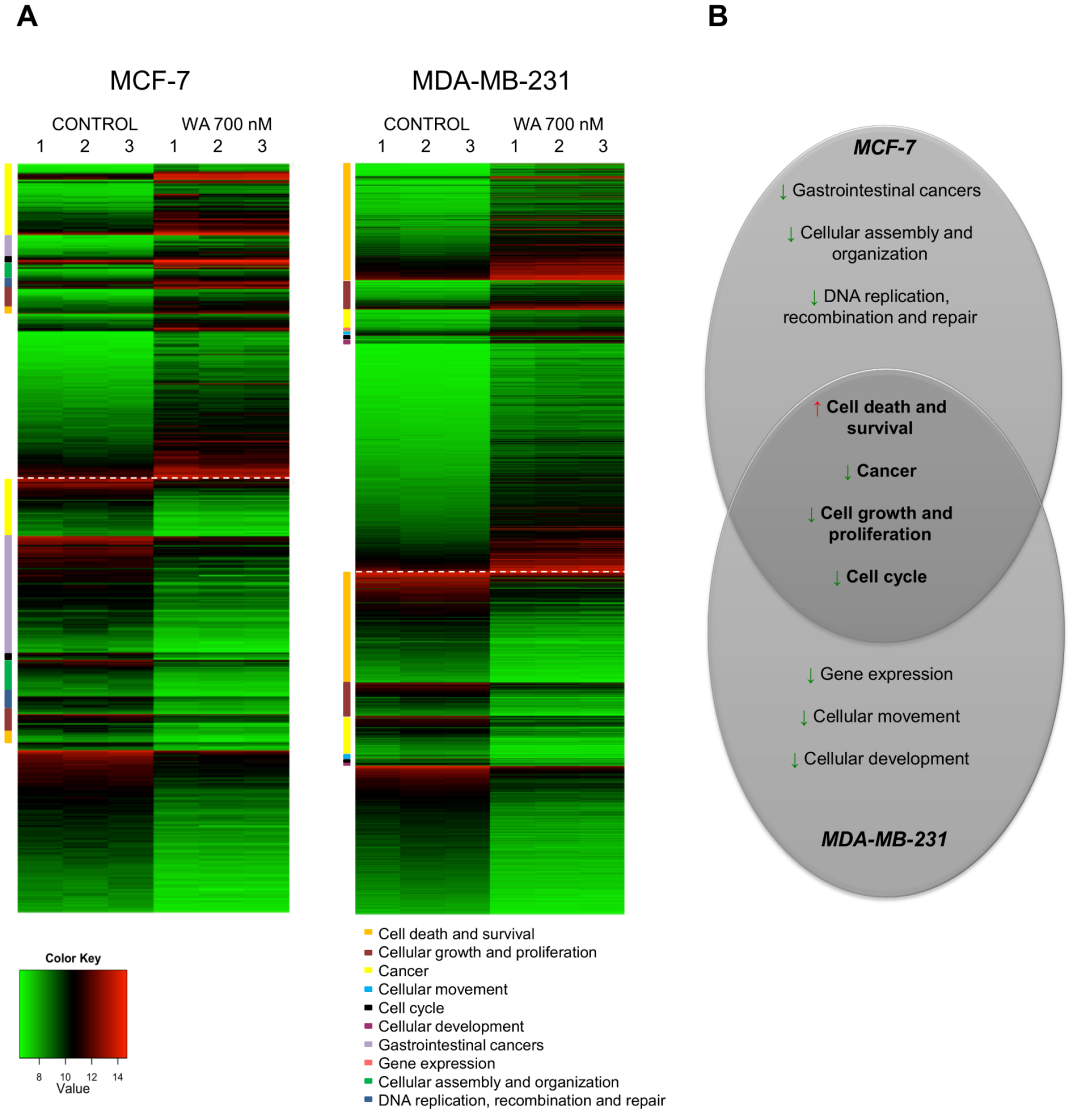
Biological processes affected by WA treatment in MDA-MB-231 and MCF-7 cells. **(A)** The heatmap represents gene expressions for control and WA 700 nM treated MDA-MB-231 and MCF-7 cells. Only genes that have a log_2_ fold change (FC)≥1 or ≤1 between control and WA-treated samples and have a p-value<0.001 are plotted. Genes that are upregulated in treated samples are plotted above the dashed line (FC≥1), those that are downregulated in treated samples are plotted below (FC≤1). Genes that belong to a specific IPA biological process are clustered and represented by a colored square next to the heatmap. Color key legend represents log_2_ normalized expression values ranging from green (low expression) to red (high expression). **(B)** Venn diagram represents common and cell line-specific IPA biological processes with predicted activation state (Red arrow represents increase of a biological process- Activation Z-score≥2; Green arrow represents decrease of a biological process- Activation Z-score≤-2).

RNA samples collected from non-invasive MCF-7 and triple negative, metastatic MDA-MB-231 cells exposed for 72 hours to WA or left untreated, were subjected to a genome-wide transcriptome profiling. The R-package “Limma” (v3.14.1) [20], [21] identified 965 downregulated and 1145 upregulated genes in MDA-MB-231 and a further 415 downregulated and 312 upregulated genes in MCF-7 cell line (including various transcript variants). The heatmaps **(** **Figure 2A** **)** represent a graphical presentation of WA-specific transcriptional changes in both cell types in three biological replicates, revealing highly reproducible and robust patterns of gene expression. Main biological processes, specified by Ingenuity Pathway Analysis (IPA), are marked with different colors and reveal common and cell type-specific responses **(** **Figure 2B** **)**. These are discussed in more detail in the next paragraph.

### WA affects four common and three cell type-specific bioprocesses in non-invasive MCF-7 and triple negative, metastatic MDA-MB-231 breast cancer cells

Differentially expressed genes (p<0.001; log_2_ ratio 1) were used as an input for the core analysis in IPA. For genes represented by multiple probes on the array (isoforms), the probe with the highest fold change was selected reducing a total number of genes in the analysis to 1580 and 499 in MDA-MB-231 and MCF-7 cells, respectively. These genes, called focus genes, were overlaid onto a global molecular network developed from information contained in the Ingenuity knowledge base. Networks of the focus genes were then algorithmically generated based on their connectivity. The Bio Functional Analysis identified the biological functions and/or diseases that were most significant to the data set. The top seven bioprocesses affected by WA were selected for further investigation, of which four were common and three revealed a cell type-specific WA response **(** **Table 1** **;** **Figure 2B** **)**.

**Table 1:** The top biological processes affected by WA in MCF-7 and MDA-MB-231 cells after 72-hour exposure to 700 nM WA.

Number of molecules	MCF-7 p-value	Biological function	MDA-MB-231 p-value	Number of molecules
202	9.31E-18-6.62E-04	Cell Death and Survival	7.11E-34-3.31E-04	543
202	7.70E-22-6.55E-04	Cellular Growth and Proliferation	4.05E-28-3.17E-04	514
266	2.91E-39-6.55E-04	Cancer	7.14E-26-3.12E-04	591
158	4.81E-28-6.55E-04	Cell Cycle	1.82E-18-2.96E-04	237
		Gene Expression	3.54E-20-2.13E-06	351
		Cellular Movement	1.03E-18-3.05E-04	307
		Cellular Development	2.67E-18-3.17E-04	215
150	2.20E-29-5.53E-04	Gastrointestinal cancers		
118	4.29E-23-6.18E-04	Cellular Assembly and Organization		
147	4.29E-23-6.55E-04	DNA Replication, recombination and repair		

The proteins encoded by these genes are involved in cell growth and proliferation, cancer progression, cell death and survival and cell cycle regulation. All commonly regulated genes by WA are presented in the heatmap **(Figure S1)**. Next, three cell type-specific WA-regulated processes, related to cellular movement, gene expression and cellular development were identified by IPA in triple negative MDA-MB-231 breast cancer cells. Inhibition of target genes in these bioprocesses predicted that WA decreased cell motility, invasion as well as epithelial-mesenchymal transition, which are all key processes of breast cancer metastasis. In MCF-7, on the other hand, cell type-specific bioprocesses included regulation of many inflammatory, gastrointestinal cancer-related genes (such as liver, colorectal, gallbladder cancers), cellular assembly and organization as well as DNA replication, recombination and repair.

In addition, upon looking at the top predicted upstream regulators of WA-responsive genes, we found nine common regulators with consistently predicted altered activation state as well as two transcription factors that were top-ranked in both cell lines according to the p-value of overlap between their known target genes and genes in the dataset **(** **Table 2** **)**. They included three cell cycle and cell proliferation controlling transcription factors, among which two transcription activators, E2F1 and FOXM1 are expected to be inhibited and one cyclin-dependent kinase inhibitor 2A (CDKNA2A) to be activated by WA treatment. Furthermore, we noted that WA administration is predicted to activate two chromatin modifying enzymes: histone demethylase JARID1B (*KDM5B*) and histone acetylase p300 (*EP300*) with transcriptional repressor and activator activity, respectively. Finally, we looked at prediction of cell line-specific transcription factor activation/inhibition. As shown in **Table 2**, WA activation of the transcription factor SPDEF (SAM pointed domain containing ETS transcription factor) shows the highest relevance in MDA-MB-231 cells, a finding which is in line with the inhibitory role of SPDEF in migration and invasion in several types of cancer [22]. On the other hand, the prediction of the WA-dependent repression of Fra-1 (*FOSL1*) transcription factor, is in line with our previous results [16] and the known role of Fra1 in cell proliferation, motility and invasiveness of breast cancer cell lines [23], [24]. This further supports the hypothesis that effectiveness of WA in triple negative breast cancer is mediated by inhibition of cell motility and invasion programs.

**Table 2:** The top upstream regulators of WA target genes ranked according to the Activation Z-score (≥ ±2) and p-value.

		MCF-7			MDA-MB-231		
Upstream Regulator	Molecule Type	Predicted Activation State	Activation z-score	p-value of overlap	Predicted Activation State	Activation z-score	p-value of overlap
TP53 *	**TR**	**Activated**	**6.158**	**1.99E-49**		**1.605**	**3.65E-32**
KDM5B	TR	Activated	4.878	5.47E-16	Activated	3.337	1.39E-07
CDKN2A	TR	Activated	4.808	1.94E-19	Activated	3.143	6.81E-06
NFE2L2	TR	Activated	3.964	9.27E-08	Activated	3.554	1.06E-03
ATF4	TR	Activated	3.261	2.28E-10	Activated	3.301	1.35E-13
EP300	TR	Activated	2.193	3.63E-05	Activated	2.012	6.12E-04
PPRC1	O	Activated	2.000	2.40E-02	Activated	2.828	2.06E-02
TRIB3	K	Inhibited	-2.950	6.66E-10	Inhibited	−3.554	3.76E-10
E2F1 *	**TR**	**Inhibited**	**-4.240**	**1.63E-42**		−**1.776**	**5.13E-14**
FOXM1	TR	Inhibited	-4.592	4.39E-24	Inhibited	−2.906	2.98E-02
TBX2	TR	Inhibited	-5.621	5.99E-32	Inhibited	−3.446	2.89E-06
SPDEF	TR				Activated	4.111	1.17E-08
CREM	O				Activated	3.636	2.49E-07
CREB1	TR				Activated	3.424	3.49E-13
TRIM24	TR				Activated	3.207	1.98E-02
SP3	TR				Activated	2.609	9.13E-03
FOXL2	TR				Activated	2.512	7.68E-03
ELF4	TR				Activated	2.449	6.80E-03
SIRT1	TR				Activated	2.281	1.89E-02
TFEB	TR				Activated	2.254	1.72E-04
ATF2	TR				Activated	2.236	5.10E-04
HOXA5	TR				Activated	2.2	3.56E-04
NOTCH3	TR				Activated	2.183	4.61E-02
NFKBIA	TR				Activated	2.134	5.43E-08
EPAS1	TR				Activated	2.05	1.68E-09
SMARCE1	TR				Activated	2	1.27E-02
DACH1	TR				Inhibited	−2	2.38E-02
FOSL1	TR				Inhibited	−2.201	1.84E-04
MLXIPL	TR				Inhibited	−2.214	3.38E-02
ERG	TR				Inhibited	−2.224	2.39E-04
MAX	TR				Inhibited	−2.229	6.47E-05
TCF3	TR	Activated	3.552	2.86E-11			
RBL1	O	Activated	3.434	2.29E-11			
RB1	TR	Activated	3.163	1.65E-24			
SMARCB1	TR	Activated	2.599	1.62E-14			
TOB1	TR	Activated	2.333	1.19E-06			
FOXO3	TR	Activated	2.225	3.40E-09			
NFATC2	TR	Activated	2.219	1.30E-01			
PPARG	LDNR	Activated	2.018	4.45E-04			
NRIP1	TR	Activated	2	1.46E-02			
MYBL2	TR	Inhibited	−2.149	1.54E-07			
NCOA3	TR	Inhibited	−2.182	1.36E-02			
SMAD7	TR	Inhibited	−2.183	5.08E-03			
FOXO1	TR	Inhibited	−2.203	8.72E-13			
IRF7	TR	Inhibited	−2.359	3.87E-03			
E2F3	TR	Inhibited	−2.433	3.19E-19			
E2F2	TR	Inhibited	−2.795	1.40E-16			
MYB	TR	Inhibited	−2.915	6.37E-05			
MYC	TR	Inhibited	−4.653	1.26E-18			

TR- Transcription regulator; O- Other; K- Kinase; LDNR- Ligand dependent nuclear receptor. * TP53 and E2F1 transcription factors were selected for further analysis based on the lowest p-values in MDA-MB-231 despite lower than ±2 Activation Z-score.

### Suppression of cell proliferation is WA-specific and related to cell cycle arrest via E2F1- and KDM5B-regulated molecular networks in both breast cancer cell lines

**Figure 3:**
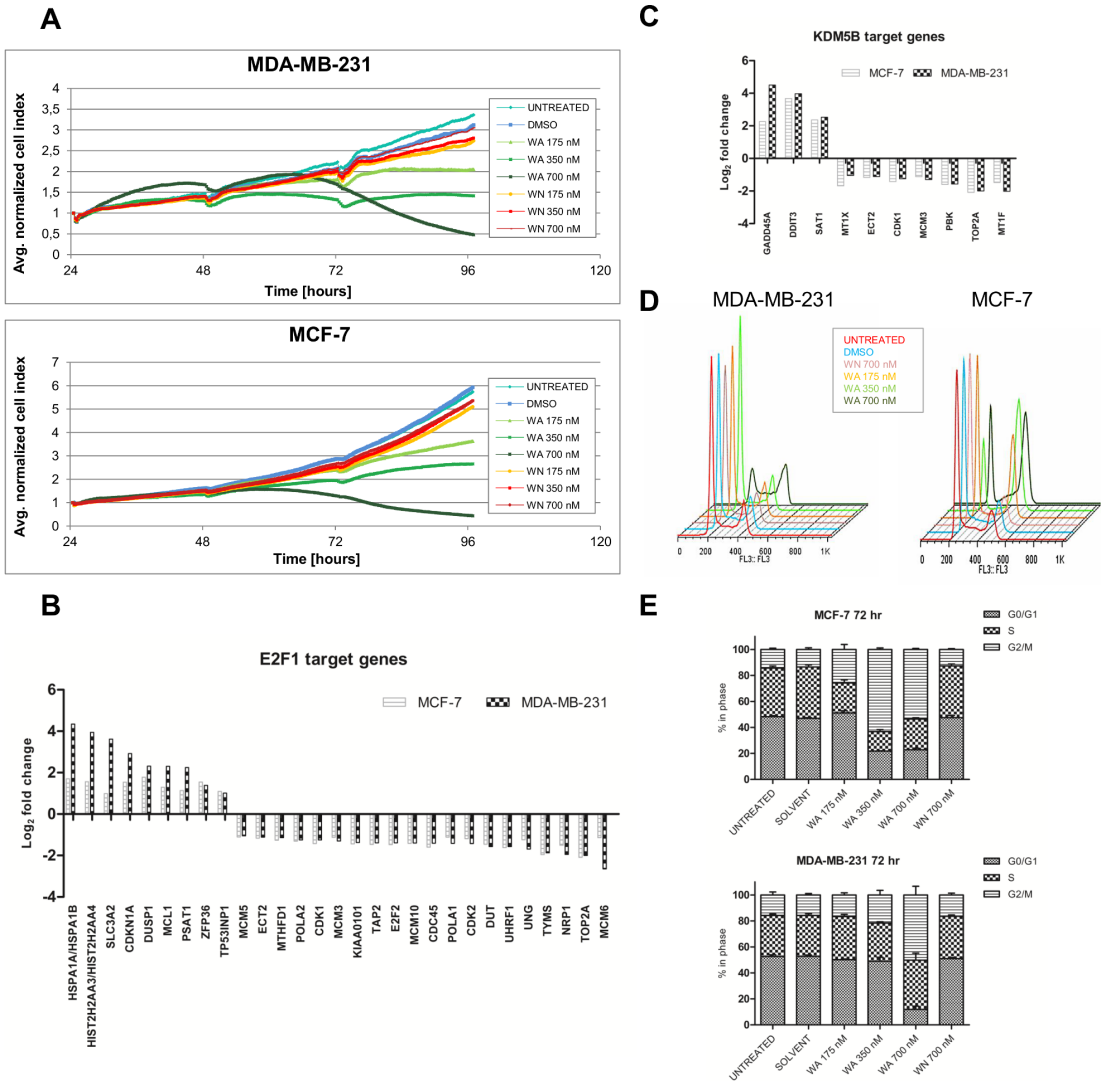
Anti-proliferative activity of WA in MDA-MB-231 and MCF-7 cells is related to cell cycle arrest. **(A)** Real-time proliferation curves as determined by *xCELLigence* RTCA. Changes in an average normalized cell index of MDA-MB-231 and MCF-7 cells left untreated or treated with solvent control, WA or WN were monitored continuously for 96 hours (24 hours before treatment + 72 hours after treatment with the compounds). Graphs represent mean values from three independent experiments starting at the time of the treatment. **(B**–**C)** Bar graphs represent mean log_2_ fold change from three independent experiments of E2F1 and KDM5B target gene expressions of 700 nM WA-treated MDA-MB-231 and MCF-7 cells relative to non-treated control samples as determined by Human HT-12 v4 BeadChip Gene Expression Array. **(D)** Analysis of the different cell cycle phases was performed by flow cytometry following Vindelov labeling procedure with propidium iodide. Representative flow histograms of MDA-MB-231 and MCF-7 cells treated for 72 hours with WA or WN compared to solvent control and untreated cells are presented. **(E)** Grouped bar graphs represent quantification of different cell cycle phases by FlowJo software using Watson Pragmatic model. Data represent (mean ± SEM) values of three independent experiments.

Sustained proliferative signaling is one of the most fundamental hallmarks of cancer. Thus, therapeutic compounds which block unlimited release of growth factors are of high therapeutic interest [25]. In this respect we performed a cell proliferation analysis using real-time, high-resolution *xCELLigence* technology. WA-mediated effects on cellular proliferation are represented as changes of average normalized cell indexes over time **(** **Figure 3A** **)**. Our results show a concentration-dependent decrease in cell proliferation in both cell lines under the influence of WA. Furthermore, in contrast to WN, WA inhibited the proliferation of breast cancer cells at low nanomolar concentrations, ranging from 175 to 700 nM. After 72-hour treatment, concentrations as low as 175 nM decreased MDA-MB-231 proliferation by (29.31±6.59)% and MCF-7 proliferation by (29.77±9.34)%. 700 nM WA almost completely abolished cell proliferation resulting in (16.66±1.52)% and (10.83±3.79)% proliferating MDA-MB-231 and MCF-7 cells, respectively. Next, using IPA analysis we searched for the possible molecular target genes responsive to WA, which could explain the above-described decreased proliferation. A role for the CDK1/cyclin B1 complex in WA-dependent growth inhibition and G2/M cell cycle arrest in breast cancer cells has already been reported [11]. These results are further supported by our transcriptome data of WA-treated MCF-7 and MDA-MB-231 cells, which reveal decreased levels of *CDK1* mRNA level as well as lowered expression of *CDK2* and *UHRF1*, regulating both G1/S and G2/M cell cycle progression. In addition, several genes encoding proteins essential for replication initiation such as mini-chromosome maintenance proteins (MCM3, MCM5, MCM6, MCM10), two DNA polymerases (POLA1, POLA2) as well as CDC45 and CDCA7 were altered, suggesting that cell cycle arrest caused by WA is not limited to G2/M phase arrest. Of special note, all abovementioned genes are downstream targets of the E2F family of transcription factors **(** **Figure 3B** **)**, which was predicted earlier as one of the top downregulated transcription regulators **(** **Table 2** **)** and experimentally observed in ChIP-seq binding data in MCF-7 cell line **(Table S1**; [26] **)**. Furthermore, JARID1B histone demethylase activated by WA treatment has been reported to associate with E2F1 target genes to silence local chromatin accessibility by removing H3K4 di- and trimethylation **(** **Figure 3C** **)**. Similarly, regulation of promoters of these genes by H3K4me2,me3 has already been shown in ChIP experiments in human mammary epithelial cells (HMEC) **(Table S2**; [26] **)**.

For further evaluation of WA-specific effects on cell cycle distribution, we performed flow cytometry after staining the cell nuclei with propidium iodide. In line with our *xCELLigence* data, we observed that only WA, but not WN, induced changes in cell cycle distribution **(** **Figure 3D** **)**. It was also clear that MCF-7 cells were more sensitive to WA-induced cell cycle arrest. Even as low as 175 nM of WA induced a significant increase in G2/M phase (p<0.05) and a decrease in S phase (p<0.0001). Higher concentrations of WA ranging from 350 to 700 nM caused a further increase in G2/M fraction (p<0.0001) and decrease in S and G0/G1 phase (p<0.0001). In contrast, MDA-MB-231 cells exhibited significant cell cycle changes only at the highest concentration of WA (700 nM), mainly related to an increase in G2/M and a decrease in G0/G1 fraction (p<0.0001) **(** **Figure 3E** **)**.

### WA, in contrast to WN, decreases invasiveness of MDA-MB-231 breast cancer cells by targeting cell motility and pro-inflammatory genes

**Figure 4:**
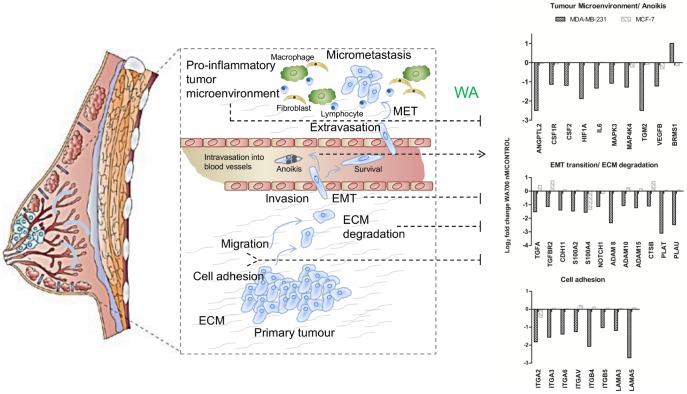
Impact of WA on several essential steps of the metastatic cascade in MDA-MB-231 cells. Graphics is a modified version of Figure 1 from [65]. Bar graphs represent mean log_2_ fold change from three independent experiments of metastasis target gene expressions of 700 nM WA-treated MDA-MB-231 and MCF-7 cells relative to non-treated control samples as determined by Human HT-12 v4 BeadChip Gene Expression Array.

Targeting metastasis in TNBC is one of the major focuses of current oncology since no agents exist that effectively block spreading of these highly invasive and often chemoresistant cells [2]. By using a well-characterized *in vitro* model for TNBC (MDA-MB-231) [27] we determined a subset of cell line-specific actors, inhibited by WA treatment, known to be implicated in cell adhesion, motility and invasion. Among them, we found genes encoding for proteases involved in invasion promoting remodeling of extracellular matrix, such as uPA (*PLAU*), ADAM metallopeptidases and cysteine proteinase- cathepsin B (CTSB), integrins which promote cell survival and migration (ITGA6, ITGB4, ITGB5, ITGAV) and several markers of epithelial-to-mesenchymal transition (TGFA, TGFBR2, CDH11, S100A2, S100A4). Some of these genes (*PLAU*, *ITGA6*) are known to be directly repressed by the SPDEF transcription factor which was predicted earlier as top activated transcription factor upon WA treatment **(** **Table 2** **)**. Additional repressed genes were *ANGPTL2*, *TGM2*, *IL-6*, *CSF1R*, *TNFSF12* as well as a key regulatory MAP kinase ERK1/2 (*MAPK3*), all known to stimulate chronic inflammatory signaling in the tumor microenvironment and to promote cancer metastasis [28]–[32]. Finally, WA increased the expression of a well established breast cancer metastasis suppressor 1 (*BRMS1*) [33] **(** **Figure 4** **)**.

**Figure 5:**
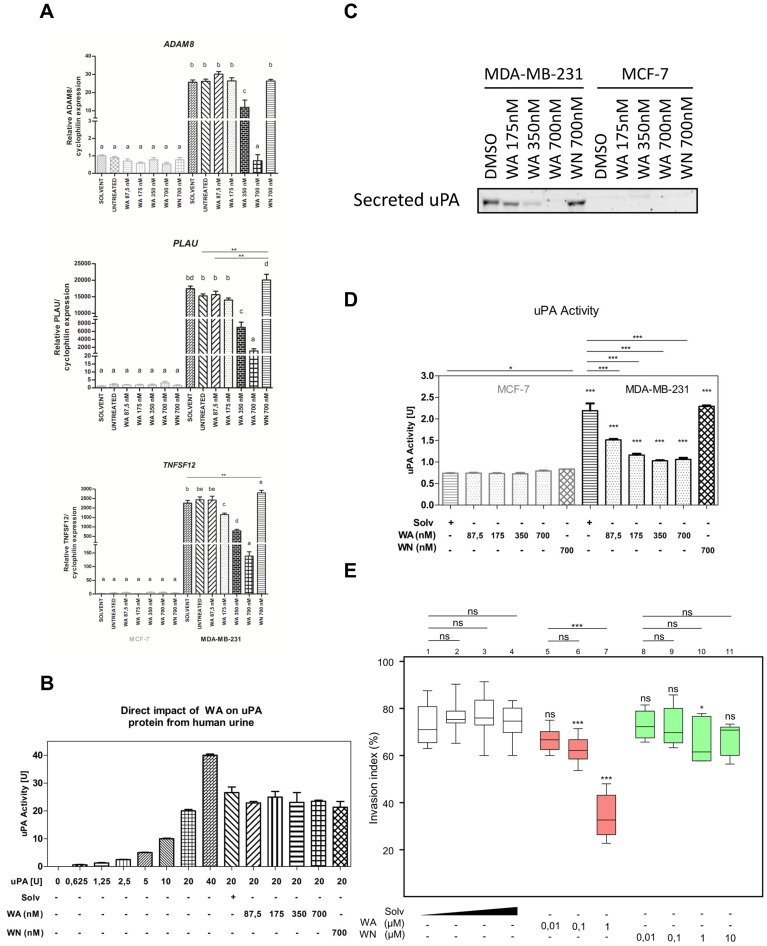
Decreased invasiveness of WA-treated MDA-MB-231 cells is associated with changed expression of ECM remodeling and pro-inflammatory genes. **(A)** Effect of WA, WN on *ADAM8*, *PLAU* and *TNFSF12* gene expression in MCF-7 and MDA-MB-231 cell lines normalized to cyclophilin housekeeping gene and relative to DMSO-treated MCF-7 sample (2^-ΔΔCt^) as determined by real-time quantitative PCR. Bar graphs represent relative mRNA (mean ± SEM) levels of three independent experiments. **(B)** Direct effect of WA, WN or DMSO on activity of uPA protein from human urine. Bar graphs represent mean ± SEM uPA activity of two independent experiments **(C)** Effects of DMSO, WN or WA at three different concentrations on uPA protein levels present in cell-conditioned medium was evaluated by Western blot. A representative blot picture of two independent experiments is shown. **(D)** The enzymatic activity of uPA in the cell-conditioned medium of MCF-7 and MDA-MB-231 cells treated with solvent, WA or WN as determined by CHEMICON colorimetric assay. Bar graphs represent mean ± SEM uPA activity of three independent experiments. **(E)** 24-hour collagen type-I invasion assay of MDA-MB-231 cells treated with DMSO, WA or WN. A box plot representing scored normalized invasion indexes of two independent experiments is shown. Within the frame, statistical significance is indicated for comparisons of treatment to the corresponding solvent condition. Above the frame, statistical significance is indicated for comparisons within a compound group.

These observations were further validated in an independent experiment, to evaluate the mRNA expression of three selected genes. These included urokinase plasminogen activator (*PLAU*) a well characterized prognostic and predictive marker for breast cancer invasion, the cell adhesion metallopeptidase ADAM8, implicated in tumor-progressive degradation of ECM proteins, and a pro-inflammatory cytokine, TNFSF12. As shown in **Figure 5A**, mRNA transcription of all these genes is elevated in aggressive MDA-MB-231 cells, compared to their levels in MCF-7 cells, and decreased dose-dependently upon WA administration. Suppression of uPA expression levels in WA-treated cells was further corroborated by measurement of its activity. We first investigated a direct inhibitory effect of WA on uPA proteolytic activity using a Chemicon assay (Merck Millipore). Chromogenic uPA substrate was incubated with 20U of uPA protein from human urine in the absence or presence of DMSO, WN or WA at several different concentrations. As shown in **Figure 5B**, neither WA nor WN, caused direct inhibition of uPA activity. On the contrary, uPA protein expression and activity in MDA-MB-231 cell-conditioned medium were decreased dose-dependently upon WA treatment **(** **Figure 5C**–**D** **)**. Of special note, this lowered protein abundance was not related to the inhibition of secretion because the intracellular levels of uPA remained very low (data not shown). To functionally assess WA's anti-invasive potential, we studied the inhibition of formation of characteristic invasive cellular extensions in the validated single-cell collagen invasion model [34] **(Figure S2)**. As depicted in **Figure 5E**, after 24 h treatment only WA exposure abrogated MDA-MB-231 invasion in a concentration-dependent manner, as compared to WN and DMSO control. Significant differences with the solvent control were reached as of 0.1 µM WA treatment. WN exposure, however, lacked any significant effect related to anti-invasive potential at concentrations ranging from 0.01 to 10 µM. These results strongly implicate that by transcriptional suppression of multiple genes involved in ECM remodeling, cell adhesion and tumor-inflammation, WA specifically diminishes invasiveness of MDA-MB-231 cells.

### WA reprograms expression of chromatin writer-reader-eraser enzymes

**Figure 6:**
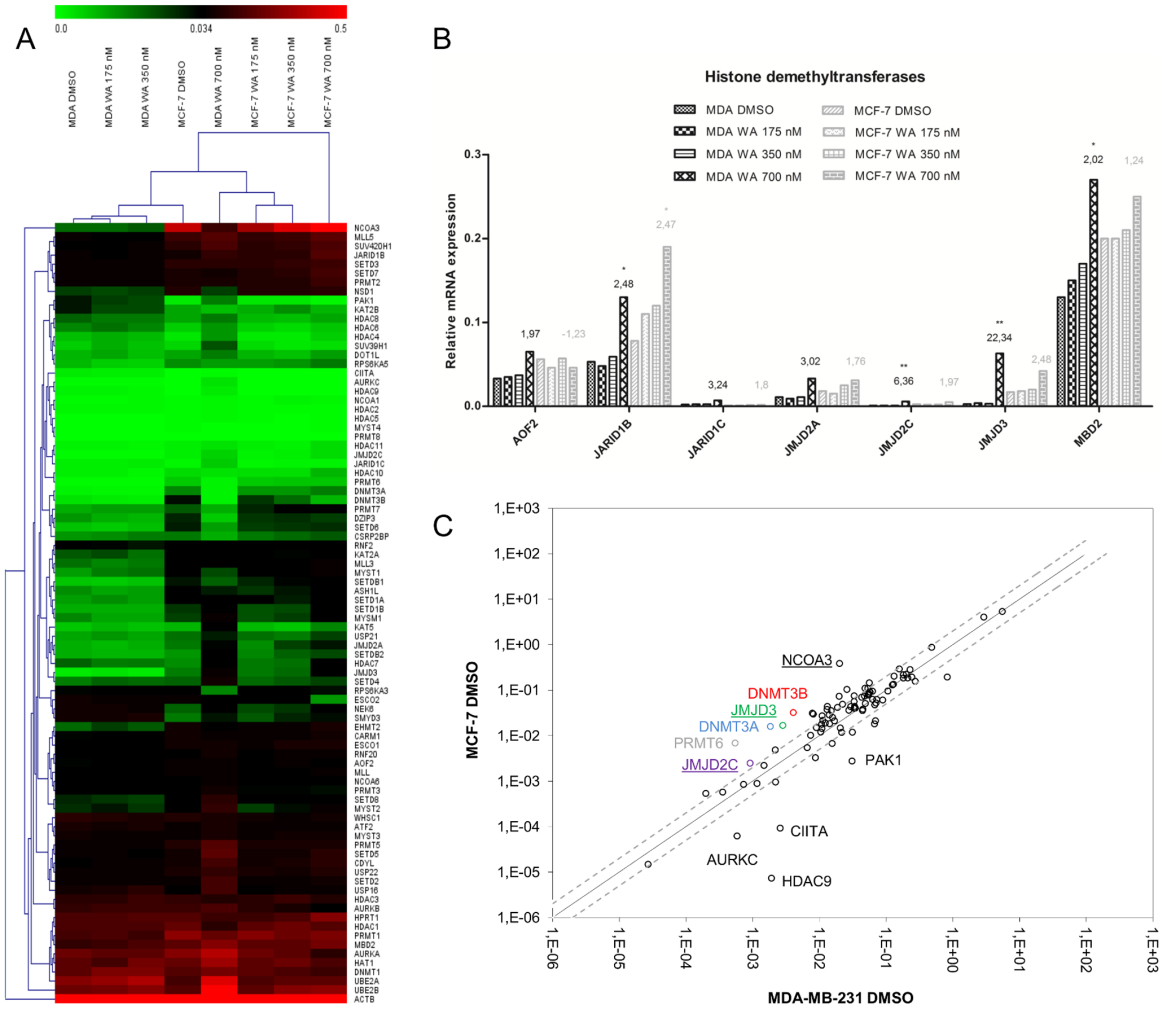
Modulation of chromatin writer-reader-eraser enzymes by WA in MDA-MB-231 and MCF-7 cells. **(A)** The heatmap represents gene expressions of 84 known or predicted genes coding for chromatin-modifying enzymes and two housekeeping genes (*ACTB, HPRT*) for control and WA-treated MDA-MB-231 and MCF-7 cells. Mean 2^−ΔCt^ values are compared pair-wise to MDA-MB-231 solvent control sample. The data represent values from at least two independent experiments. Color key legend above the heatmap represents 2^−ΔCt^ normalized expression values ranging from green (low expression) to red (high expression). **(B)** Bar graphs representing mean 2^−ΔCt^ normalized expression values of genes encoding for histone demethyltransferases from at least two independent experiments. Fold changes between control (DMSO) and 700 nM WA-treated samples are indicated above the corresponding bars. Significantly upregulated genes are marked with asterisks. **(C)** The scatter plot of the mean 2^−ΔCt^ normalized expression values of each gene in the control sample (MDA-MB-231 DMSO) versus the test sample (MCF-7 DMSO). The most differentially expressed genes are labeled next to the corresponding dot. WA-regulated genes are underlined. The black line indicates fold change 2^−ΔΔCt^ of 1. The dashed, gray lines indicate desired fold-change in gene expression threshold, here defined as 2.

Because IPA analysis predicted JARID1B and p300 chromatin enzymes as top transcriptional regulators of WA-responsive gene expression, we wanted to explore whether WA changes expression of additional chromatin-modifying enzymes. It is known that deregulated or mutated chromatin enzymes and miswritten epigenetic marks strongly contribute to cancer etiology [35] and the therapeutic interest in modulation of these marks is growing. In an independent set of experiments, we performed Human Epigenetic Chromatin Modification Enzyme qPCR Array to compare the expression of 84 well-known chromatin reader, writer or eraser enzymes in both breast cancer cell types exposed to solvent control or WA. The heatmap shown in **Figure 6A** represents mean 2^−ΔCt^ values of gene expression of at least two independent experiments compared to MDA-MB-231 control sample. The highest concentration of WA (700 nM) triggered the most pronounced changes in cofactor gene expression in MDA-MB-231 cells **(Figure S3)**, which after the treatment clustered together with samples of MCF-7 epithelial-like phenotype **(** **Figure 6A** **)**. Besides modulation of SET domain proteins with histone methyltransferase activity as well as histone ubiquitin ligases, WA predominantly increased transcription of jumonji domain enzymes. In the MCF-7 cell line, JARID1B (*KDM5B*) was among two histone demethylases whose expression increased by more than two-fold **(** **Figure 6B** **)**. Other groups of chromatin modifiers regulated in MCF-7 included Aurora kinases. In general, the number of WA (up/down) regulated epigenetic cofactors in MCF-7 cells was smaller than in MDA-MB-231 cells, implying that the epigenetic plasticity of MCF-7 cells is more restricted than in MDA-MB-231 cells. All transcripts which are significantly up- or downregulated in either one or both cell lines upon 700 nM WA treatment are listed in **Table 3**. In addition, **Figure 6C** represents a comparison of basal levels of epigenetic modulators in non-invasive MCF-7 and the highly aggressive MDA-MB-231 cells. Underlined genes (JMJD3, JMJD2C, NCOA3) change their expression after WA administration in the direction towards the less aggressive MCF-7 cell line, which may imply their role in invasiveness or reflect the difference between cancer cells originating from a primary tumor versus a metastatic tumor site. The exact interplay of multiple chromatin enzymes in pro/anti-metastatic activities in breast cancer however requires further investigation.

**Table 3:** Significantly differently expressed chromatin and DNA modifying enzymes as determined by Human Epigenetic Chromatin Modification Enzyme qPCR Array.

		MDA-MB-231 WA 700 nM/MDA-MB-231 DMSO		MCF-7 WA 700 nM/MCF-7 DMSO	
Cofactor group	Gene	Fold change	p-value	Fold change	p-value
**DNA methyltransferases**	DNMT3B	ns*	ns	−3.13	1.63E-03
**Histone acetyltransferases**	CDYL	3.41	4.57E-02	ns	ns
	MYST2	4.54	3.22E-02	ns	ns
	NCOA3	7.65	2.33E-02	ns	ns
	ESCO2	ns	ns	−3.79	4.91e-03
**Histone methyltransferases**	PRMT5	3.06	1.71E-02	ns	ns
	SMYD3	−2.17	9.19E-04	ns	ns
**SET domain proteins (Histone methyltransferase activity)**	SETD2	2.57	2.59E-02	ns	ns
	SETD3	2.00	2.82E-02	ns	ns
	SETD4	4.52	1.16E-03	ns	ns
	SETDB2	3.60	1.05E-02	ns	ns
	SUV420H1	3.74	2.15E-02	ns	ns
**Histone demethyltransferases**	JARID1B	2.48	1.57E-02	2.47	1.14E-02
	JMJD2C	6.36	9.15E-03	ns	Ns
	JMJD3	22.34	9.14E-03	ns	Ns
	MBD2	2.02	3.18E-02	ns	Ns
**Histone ubiquityn ligases**	MYSM1	3.41	8.30E-03	ns	Ns
	UBE2B	3.45	6.60E-03	ns	Ns
	USP16	2.35	3.37E-03	ns	Ns
	USP21	2.47	3.56E-02	ns	Ns
**Histone kinases**	AURKA	ns	ns	−2.37	9.98E-03
	AURKB	ns	ns	−3.75	4.36E-02
	AURKC	6.78	2.21E-02	4.60	1.71E-02
	RPS6KA3	−2.04	4.00E-02	ns	Ns

* **ns- not significant.**

## Discussion

The number of reports describing anticancer properties of WA *in vitro* and more recently *in vivo* are rapidly growing [5], [36]. Hence, a better understanding of its molecular mechanisms in cancer cells is of primordial interest. Our data provide further insight into multiple pathways targeted by WA in *in vitro* breast cancer cell models with varying metastatic potential. Via microarray transcriptome profiling of MCF-7 and MDA-MB-231 cells exposed to systemic achievable concentrations of WA, we identified common as well as cell line-specific target genes. In the context of common cancer hallmarks affected by WA treatment, we show here that the growth inhibitory potential of WA is mediated mainly by cell cycle arrest. A real-time *xCELLigence* proliferation assay as well as flow cytrometric cell cycle analysis revealed a concentration- dependent decrease in cell proliferation rate **(** **Figure 3A** **)**, which correlated with an increased cell population stalled in G2/M phase **(** **Figures 3D**–**E** **)** and decreased cell viability in both cell lines **(** **Figure 1A**–**D** **)**. Similarly to Stan et al. we confirmed that WA decreased expression of cyclin-dependent kinase 1 (CDK1), a regulator of G2/M transition [11]. However, longer WA treatment resulted in additional activation of cyclin-dependent kinase inhibitor 1A, which coincided with downregulation of CDK2. In addition, numerous S phase regulatory genes were also co-regulated in both cell lines, but a significant decrease in S phase could only be detected in MCF-7 cells **(** **Table 1** **,** **Figure 2B** **,** **Figure 3E** **)**. We show here, for the first time, that a remarkable number of cell cycle regulatory genes modulated by WA in both cell lines represent E2F1 target genes **(** **Figure 3B** **)**. The E2F1-mediated regulation of these genes was predicted by IPA analysis **(** **Table 2** **)** and further supported by ChIP experiments in MCF-7 cells as specified by cSCAN analysis **(Table S1)**. However, further functional assays are required to confirm the direct regulation of E2F1 transcription factor activity by WA.

In addition, our study was designed to identify cell line-specific regulators which could provide new mechanistic insights into therapeutic strategies in highly aggressive, triple negative breast cancer. We identified three cell line-specific processes regulated by WA, of which the most pronounced was regulation of cell movement. This category included genes related to cell motility, extracellular matrix remodeling, adhesion and invasion, as depicted in **Figure 4**. The multitude of WA targets as well as the modulation of cytoskeleton and extracellular matrix proteins suggested possible organism-wide debilitating effects of WA, as previously proposed by Grin and colleagues [14]. Indeed, as shown in our transcriptome analysis, WA modulates expression of several genes involved in basic physiological processes. However, when comparing expression data from both cell lines, it is apparent that WA-mediated inhibition of integrins and laminins is selective towards aggressive MDA-MB-231 cells. It has previously been reported that aggressive breast cancers, despite the decreased basement membrane deposition, constitutively overexpress some laminins and integrin receptors which functionally contributes to the motile and invasive phenotype of these cells. Consequently, targeting their overexpression could be of great therapeutic interest [37]–[40]. Of special note, the selective integrin antagonist cilengitide has passed the Phase II clinical trials and is now being tested in a Phase III trial in patients with glioblastoma [41]. Furthermore, WA caused concentration-dependent inhibition of mRNA transcription of selected ECM-remodeling proteases (ADAM8, ADAM10, CTSB and PLAT, uPA). The role of the abovementioned genes in invasion and metastasis is well documented [33], [42]–[44] and further supported by very high basal expression of *ADAM8, TNFSF12, PLAU* genes in invasive MDA-MB-231 cells as compared to non-invasive MCF-7 cells **(** **Figure 5A** **)**. The regulation of invasion-modulating genes by WA but not by WN was mirrored by our single cell collagen invasion assay as well as by uPA activity and protein detection assays of MDA-MB-231 cells, in which we could show a clear anti-invasive biological effect of WA but lack of effect for WN. Further insight into the regulation of uPA revealed that WA reduced its protein expression and activity via transcriptional control mechanism, as neither secretion nor the intrinsic activity were abrogated by WA treatment **(** **Figures 5B**–**E** **)**. Both uPA and ADAM proteases are recognized as valuable biomarkers for cancer prognosis and a number of selective inhibitors of these proteins (INCB7839, INCB3619) [42], [45], [46] have revealed promising anti-tumor responses in phase I/II trials. The observation that WA treatment downregulates mRNA expression of ADAM8 and uPA, and negatively affects uPA protein levels and enzymatic activity, further strengthens the potential therapeutic effect of WA in cancer therapy. Moreover, WA decreased epithelial-to-mesenchymal transition regulatory genes **(** **Figure 4** **)**. In line with Thaiparambil et al., we confirmed that invasion inhibiting doses of WA after 24 h exposure were as low as 100 nM and not related to cytotoxic effects. The same authors have shown that this initial anti-invasive response may be related to vimentin ser56 phosphorylation and its perinuclear disassembly. However, short term WA treatment did not result in significant changes in typical EMT markers (E-cadherin, N-cadherin), leading the authors to conclude that WA does not change the mesenchymal phenotype of MDA-MB-231 cells [13]. On the contrary, in our experimental set up WA decreased EMT-related components of the TGF-β signaling pathway (TGFA, TGFBR2, CDH11 [47], [48]), as well as TGM2, HIF1A, several TNFSF family members and ANGPTL2, which stimulate tumor promoting pro-inflammatory responses and a hypoxic microenvironment. Inhibition of invasion was enforced by upregulation of well characterized breast cancer metastasis suppressor 1 (BRMS1), which was shown to regulate various steps of the metastatic cascade including invasion, cell survival at secondary sites and colonization of distant organs [33], [49].

Finally, based on previous findings revealing chromatin-dependent transcriptional regulation of IL-6 by WA in MDA-MB-231 cells [16], we further explored the regulation of chromatin writer-reader-eraser enzymes in WA-treated cells. Nowadays, it is becoming clear, that misregulation of histone modifications and DNA methylation catalyzed by these enzymes actively contributes to human cancer, or in particular cancer cells chemoresistance, proliferation and metastasis [35], [50], [51]. As such, profiling of 84 known epigenetic enzymes in MDA-MB-231 and MCF-7 cells exposed to three doses of WA as compared to solvent control indeed revealed modulation of several classes of epigenetic enzymes in both cell lines. However, MDA-MB-231 cells exposed to 700 nM WA were subjected to the largest expression changes upon WA treatment and clustered together with samples of MCF-7 with a non-metastatic, epithelial-like phenotype as depicted in the heatmap **(** **Figure 6A** **)**. Moreover, having a closer look at different classes of epigenetic modulators, we could confirm WA-responsive regulation of JARID1B (*KDM5B*) in both cell lines, involved in regulation of E2F1 target genes, in line with the pathway analysis of our microarray data [52]. In MDA-MB-231 cells, we observed additional WA-specific upregulation of other members of jumonji domain proteins, JMJD3 (KDM6B) and JMJD2C **(** **Figure 6B** **)**. However, conflicting data have been published related to either tumor-promoting or -suppressing activities of JARID1B, JMJD3 and JMJD2C, suggesting that the overall effect in cancer may be context-dependent and rely on concerted action of multiple epigenetic enzymes and/or combinatorial posttranslational modifications of nucleosomes and transcription factors [53]–[59]. Finally, with exception of decreased expression of DNMT3B in MCF-7 cells, we could not support a major role for WA in changing expression of DNMTs, as recently reported by Mirza et al [19]. Further studies need to clarify whether WA can elicit stable gene specific DNA methylation changes besides dynamic regulation of chromatin remodeling and nucleosome accessibility.

In conclusion, the steroidal withanolide WA, isolated from *Withania somnifera* (Ashwagandha) has become an increasingly recognized phytomedicinal anticancer compound worldwide [6]. Despite intensive efforts and development of various chemotherapeutic agents in the clinic, the efficacy of various therapies is still limited by the heterogeneity of cancer cells and complex tumor-stroma microenvironment interactions, which ultimately result in clonal selection of a drug-resistant cell population [60]. In this respect, natural medicinal compounds gained renewed interest as chemotherapeutic agents to prevent or overcome therapy resistance, due to their capacity to target multiple cancer hallmarks, including proliferation, cell death resistance, replicative immortality, invasion and metastasis, angiogenesis and tumor-promoting inflammation [61], [62]. By applying a pathway-based transcriptome analysis of WA effects in non-aggressive versus triple negative metastatic breast cancer cells, we identified various novel molecular targets related to its anti-proliferative, anti-metastatic and epigenetic mode of action. In summary, we demonstrate that WA affects several clinically relevant targets in breast cancer cells and have identified WA-dependent inhibition of the uPA pathway as a novel mechanism underlying its potent anti-metastatic activities. Remarkably, the closely related withanolide WN did not reveal any anticancer or anti-metastatic activity at similar concentrations. The efficacy of WA is clearly concentration-dependent and attenuation of metastasis in MDA-MB-231 cells could be observed at the lowest concentrations and might be related to multifocal inhibition of signaling networks involving ECM remodeling i.e. uPA signaling, pro-inflammatory tumor cytokines and cell adhesion. Altogether, our results suggest that WA based therapeutic strategies hold promise for further (pre)clinical development to defeat aggressive metastatic breast cancer.

## Materials and Methods

### Reagents

Withaferin A and Withanone (purity≥97%, purchased from Altavista Phytochemicals Pvt Ltd.) were dissolved in DMSO (Hybri-Max, suitable for cell culture, Sigma-Aldrich, St. Louis, MO, USA) to a stock solution of 20 mM and further diluted in complete growth medium to a final concentration immediately before use. GelRed™ Nucleic Acid Stain in water (10 000× stock) was purchased from Biotium (Hayward, CA, USA). DTT was purchased from Sigma-Aldrich (St. Louis, MO, USA) and a stock solution of 1 mM was prepared in water. Anti-uPA antibody (sc-14019) was purchased from Santa Cruz Biotechnology (Santa Cruz, CA, USA).

### Cell lines and cell culture

The MDA-MB-231 and MCF-7 cell lines, purchased from American Type Culture Collection (Manassas, VA, USA), were cultivated in high glucose (4,5 g/L) Dulbecco's Modified Eagle Medium with 10% Fetal Bovine Serum, 2 mM L-Glutamine, 1 mM Sodium Pyruvate MEM, 50 IU/mL Penicillin and 50 µg/mL Streptomycin. The MDA-MB-231 cell line was additionally supplemented with 1% MEM Non-Essential Amino Acids. Each cell line was maintained at 37°C in the humidified atmosphere containing 5% CO_2_ until 80–90% confluence. Both cell lines have successfully been authenticated by short tandem repeat (STR) profiling (Cell ID System, Promega, Madison, WI, USA) according to the manufacturer's instructions. Prior to experiments with WA or WN we evaluated cell growth properties, by means of *xCELLigence* system (Roche, Penzberg, Germany), to determine the optimal seeding density for a long-term experiment, which was 2 x 10^4^ viable MDA-MB-231 cells/cm^2^ and 5,3 x 10^3^ viable MCF-7 cells/cm^2^. Suitable amount of cells was plated in an appropriate cell culture flask/plate 24 hours before the treatment. Every 24 hours, compounds or DMSO controls (final concentration 0,0035% if not otherwise stated) diluted in fresh medium were added for a total time of 72 hours. At that time point, cells were harvested with 0.05% Trypsin/EDTA solution, washed with 1× PBS and collected for further analysis. All cell culture reagents were purchased from Life Technologies (Praisley, UK).

### Flow cytometry

#### Cell viability

Cell viability was determined by flow cytometry through addition of 1× GelRed™ Nucleic Acid Gel Stain (Biotium, Hayward, CA, USA). MDA-MB-231 and MCF-7 cells were plated in 24-well plates and treated with 6 different concentrations of WA (0.0875, 0.175, 0.35, 0.7, 1.4, 2.8 µM) or mock control (0.014% DMSO) as described above. After the indicated time point, cell growth medium, PBS wash solution as well as harvested cells were collected and centrifuged for 5 min at 250 x g. The cell pellet was resuspended in 1 mL of PBS containing 5% FBS. For each concentration, three replicates were performed and directly analyzed using an Epics XL-MCL analytical flow cytometer (Beckman Coulter, Fullerton, CA, USA). The mean value of at least three independent experiments was calculated. IC_50_ concentrations were estimated for 72 h treatments using GraphPad Prism version 5.00 for Windows, modeling a dose response curve starting from 100% viability in DMSO controls to 0% viability at the highest concentrations of WA. The hill slope was not fixed.

#### Cell cycle analysis

Cell cycle distribution was assessed according to the Vindelov method, as previously described [63]. After 72 hours treatment cells were harvested, washed with PBS and two aliquots of approximately 1 x 10^6^ cells were used for subsequent staining procedure in each of three independent experiments. Epics XL-MCL analytical flow cytometer (Beckman Coulter, Fullerton, CA, USA) was used to measure the DNA content and the data was analyzed in FlowJo software (©Tree Star, Inc., Ashland, OR, USA) using Watson pragmatic model. One-way ANOVA with Tukey's post test was performed using GraphPad Prism version 5.00 for Windows (GraphPad Software, La Jolla California, USA) to determine statistically significant differences in the percentages of tumor cells in each cell cycle phase (mean ± SEM) between samples.

### Real-time monitoring of cell proliferation using *xCELLigence* (RTCA)

MDA-MB-231 and MCF-7 cell growth was continuously monitored in 15 min intervals for a total time of 96 h using the *xCELLigence* RTCA DP instrument (Roche). Background impedance signal was measured following 30 min incubation with 100 µL of a suitable cell culture growth medium per well. The final volume per well was adjusted to 150 µL by adding 50 µL of cell culture suspension in a complete growth medium containing a suitable amount of cells. 24 hours after seeding, DMSO control or WA at 3 different concentrations (0.175, 0.35, 0.7 µM) were added in a total volume of 150 µL. An additional control contained untreated cells. For each concentration, two replicates on an E-plate 16 were performed. The impedance signal was analyzed by normalizing data of each well to the last measurement before the compound treatment. CI_(normalized)_ = CI_time x_/CI_norm time_. The normalized cell indexes of three independent experiments were used for graphical result representation and cell proliferation measurement.

### Invasion assay

Single cell collagen invasion assays were performed as previously described [34]. In brief, MDA-MB-231 cells were seeded on a collagen type-I matrix and treated with compounds or solvent for 24 hours, after which the situation was documented via phase-contrast microscopy using a Leica DMI3000B microscope, a DFC 340 FX camera Twain version 7.4.0.0 and Leica application Suit (LAS) v3.8.0 software. Invasiveness was scored by assessing the protrusion of cellular extensions into the collagen of at least 7 pictures per condition. The number of invasive cells was normalized to the total number of cells and expressed as the invasion index. Statistical analysis was performed, using a Mann Whitney U test, showing significance of selected pair-wise comparisons. Values of p<0.05 were considered significant.

### RNA extraction and microarray processing

Total RNA from non-treated controls and WA-treated (700 nM) MDA-MB-231 and MCF-7 cells from three independent experiments was isolated using 1 mL of TRI Reagent (Sigma-Aldrich, St.Louis, MO, USA) per 5 x 10^6^ cells and further proceeded according to the manufacturer's protocol till the step of phase separation. After transferring the aqueous phase to a new 1.5 mL micro tube, an equal amount of 70% ethanol was added and samples were further purified on RNeasy spin columns (Qiagen, Hilden, Germany) according to the manufacturer's protocol. Following extraction and concentration measurement (NanoDrop 1000, Thermo Scientific, Waltham, MA, USA) total RNA was quality controlled on a Bio-Rad experion (Bio-Rad, Hercules, CA, USA). 500 ng of total RNA was amplified using the Illumina TotalPrep RNA Amplification kit (Life Technologies, Carlsbad, CA, USA). Briefly, RNA was reverse transcribed using T7 oligo(dT) primers, after which biotinylated cRNA was synthesized through an *in vitro* transcription reaction. 750 ng of amplified cRNA was hybridized to a corresponding array of a HumanHT12 beadchip (Illumina, San Diego, CA, USA). In total, 12 array hybridizations were performed. The beadchip was incubated for 18 hours at 58°C in a hybridization oven under continuous rocking. After several consecutive washing steps (see manufacturer's protocol), bead intensities were read on an Illumina Iscan.

### Microarray data analysis

Raw data intensities were read in R using the “beadarray” package (v2.8.1) [64]. Intensities were quantile normalized and differential gene expression between samples was estimated using “limma” (v3.14.1) [20], [21]. Resulting p-values were corrected for multiple hypothesis testing using the Benjamini Hochberg procedure. Next to estimating gene expressions, euclidean distances between samples were calculated and used as a distance metric in a hierarchical cluster analysis.

Pathway analysis was performed in the Ingenuity Pathway Knowledge Base (Ingenuity® Systems, www.ingenuity.com, Redwood City, CA, USA) according to the instructions provided. A fold change cut-off of 2 as well as false discovery rate of 0.1% were set to identify genes whose expression was significantly differentially regulated. The IPA automatically removed the duplicate or unknown gene names from analysis resulting in lower number of genes as compared to the total number of transcripts in the microarray. Fischer's exact test was used to calculate a p-value determining the probability that each biological function and/or disease assigned to that data set is due to chance alone. Raw array data were uploaded to the Gene Expression Omnibus (GEO) database and have accession number: GSE53049 (http://www.ncbi.nlm.nih.gov/geo/query/acc.cgi?acc=GSE53049).

### Reverse transcription (RT)- PCR and real-time quantitative PCR

1 µg of total RNA was reverse transcribed using oligo dT primers (Life Technologies, Praisley, UK) and M-MLV Reverse Transcriptase (Promega, MA, USA). Relative mRNA levels of genes of interest were quantified by real-time quantitative PCR reaction on ABI Prism 7300 (Applied Biosystems) and normalized against cyclophilin housekeeping gene. Sequences of cDNA-specific primers are available upon request. The 2^−ΔΔCt^ method was used for calculation of relative expression levels between samples. Statistical analysis was performed using GraphPad Prism version 5.00 for Windows (GraphPad Software, La Jolla California, USA). Relative gene expression values of three independent experiments (mean ± SEM) are represented on the bar graphs. The bars in the graphs marked with different letters are significantly different (p<0.0001 unless otherwise stated) as determined by one-way ANOVA (Tukey Multiple Comparison test).

### Western blotting

MDA-MB-231 and MCF-7 cells were treated with WA, WN or DMSO control as previously described. At the end of the incubation time cell-conditioned medium was collected. Protein concentrations were determined by the DC protein assay (Bio-Rad, CA, USA). Samples from cell-conditioned media were prepared by taking volume of supernatant with equal amount of protein, addition of distilled water to final volume of 20 µl and addition of 5 µl 5× Laemli sample buffer (300 nM Tris pH 6,8, 10% SDS, 50% glycerol, 0,05% Bromophenol Blue, 25% β-mercaptoethanol). Proteins were separated on 8.5% SDS-PAGE, and transferred onto a nitrocellulose membrane. Non-specific binding sites on the membrane were blocked with a mixture of 50% Licor blocking buffer (Licor, Lincoln, NA, USA)/50% TBS containing 0.2% Tween-20 for 1 hour. Afterwards membranes were incubated with uPA recognizing primary antibody and visualized with fluorophore-coupled secondary antibody. Additional loading control for samples from cell-conditioned media was performed by staining the nitrocellulose membranes with 0,1% ponceau red stain. Detection was performed by use of the Odyssey Imaging System (Licor, Lincoln, NA, USA).

### uPA enzyme activity assay

We examined the enzymatic activity of uPA in 160 µL of cell-conditioned medium of the cells treated with solvent controls, WA or WN at indicated concentrations, using the CHEMICON assay (EMD Millipore, Merck, Darmstadt, Germany). This colorimetric assay measures a cleavage of a chromogenic uPA substrate, which results in a colored product, detectable by optical density at 405 nm. The standard curve was created by serial dilutions of known amounts of uPA protein from human urine. Moreover, 20U of the uPA protein was pre-incubated for 30 minutes with DMSO, WN or different concentrations of WA to probe a direct inhibitory effect of the compounds on uPA protein activity. Absorbance was read on 2102 EnVision Multilabel Plate Reader (PerkinElmer, Waltham, MA, USA) following 6 h 30 min incubation at 37°C. One-way ANOVA with Tukey's post test was performed to determine statistically significant differences in the activity (mean ± SEM) between samples.

### Human Epigenetic Chromatin Modification Enzyme qPCR Array

The Human Epigenetic Chromatin Modification Enzyme RT^2^ Profiler™ PCR Array (PAHS-085A) (SABiosciences, Qiagen) was used to determine the expression levels of 84 key genes coding for chromatin modifying enzymes. 1 µg of total RNA, treated with RNase-Free DNase Set (Qiagen), was used for further processing according to the manufacturer's protocol. cDNA synthesis was performed by RT^2^ First Strand Kit (C-03). The RT^2^ SYBR Green ROX™ qPCR Mastermix was used for preparing the experimental cocktail according to the protocol and 25 µL was dispensed to each well of 96-well PCR Array plate. Relative mRNA levels of genes of interest were quantified by real-time quantitative PCR reaction on an ABI Prism 7300 (Applied Biosystems, CA, USA) and normalized against selected housekeeping genes (HPRT, ACTB). An Excel-based RT^2^ Profiler PCR Array Template (V3.3) (http://www.sabiosciences.com/pcrarraydataanalysis.php) was used for statistical analysis (paired T-test) and fold change quantification of the samples treated with WA as compared to DMSO controls in both cell lines. A gene was considered not detectable when Ct>35. Moreover, Ct was defined as 35 for the ΔCt calculation when the signal was under detectable limits. Fold-change and fold-regulation values>2 were indicative of upregulated genes; fold-change values <0.5 and fold-regulation values <-2 were indicative of downregulated genes.

## Supporting Information

Figure S1 **WA target genes co-regulated in MCF-7 and MDA-MB-231 cells.** The heatmap represents the expression of 202 genes regulated by WA (Fold change ≥2, p<0.001) in a highly consistent manner between MCF-7 and MDA-MB-231 cells. 114 genes are up-regulated and 85 genes are down-regulated in both cell lines with only 3 commonly regulated genes showing inconsistent regulation. Expression values are log transformed and expressed as row z-score (difference between expression value and average expression value of the gene divided by gene standard deviation).
(TIF)

Figure S2 **Representative phase-contrast microscopy pictures depicting decreased MDA-MB-231 cell invasion following WA, but not WN, treatment as determined by a 24-hour collagen type-I invasion assay.** The quantity of solvent in ‘Solv’ was matched with the amount of DMSO in the corresponding treatment. Invasive cells are indicated with white arrows; non-invasive cells are indicated in the middle panel.
(TIF)

Figure S3 **Concentration-dependent regulation of chromatin modifying enzyme gene expression by WA in MDA-MB-231 and MCF-7 cells.** The scatter plots of the mean 2^−ΔCt^ normalized expression values of each gene in the control samples (MDA-MB-231 DMSO, MCF-7 DMSO) versus the test samples (set-ups with WA) reveal the largest epigenetic plasticity of MDA-MB-231 cells exposed to the highest WA concentration (700 nM). The black line indicates fold change 2^−ΔΔCt^ of 1. The dashed, gray lines indicate desired fold-change in gene expression threshold, here defined as 2.
(TIF)

Table S1 **A list of experimentally validated ChIP- sequencing data for genes regulated by E2F1 transcription factor in MCF-7 breast cancer cells.**
(DOCX)

Table S2 **A list of experimentally validated ChIP- sequencing data for genes regulated by H3K4me2,me3 in Human Mammary Epithelial Cells (HMEC).**
(DOCX)
