# Supplementary figures and images for: Pharmacological Levels of Withaferin A (Withania somnifera) Trigger Clinically Relevant Anticancer Effects Specific to Triple Negative Breast Cancer Cells
Source: PLoS One. 2014 Feb 3;9(2):e87850. doi: 10.1371/journal.pone.0087850 (PMC3912072; doi:10.1371/journal.pone.0087850)

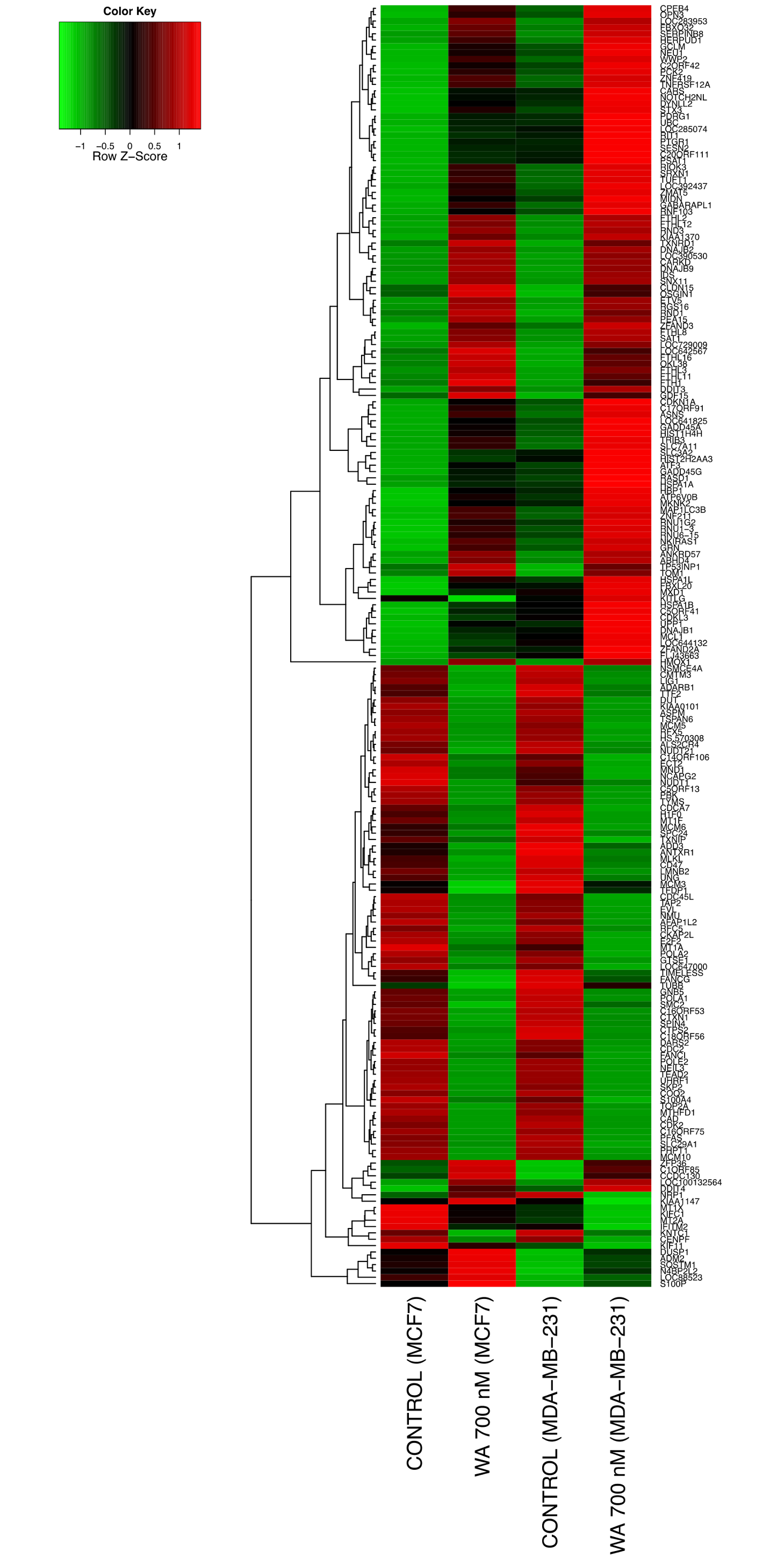

Supplement: Figure S1 — WA target genes co-regulated in MCF-7 and MDA-MB-231 cells. The heatmap represents the expression of 202 genes regulated by WA (Fold change ≥2, p<0.001) in a highly consistent manner between MCF-7 and MDA-MB-231 cells. 114 genes are up-regulated and 85 genes are down-regulated in both cell lines with only 3 commonly regulated genes showing inconsistent regulation. Expression values are log transformed and expressed as row z-score (difference between expression value and average expression value of the gene divided by gene standard deviation). (TIF) [file pone.0087850.s001.tif]

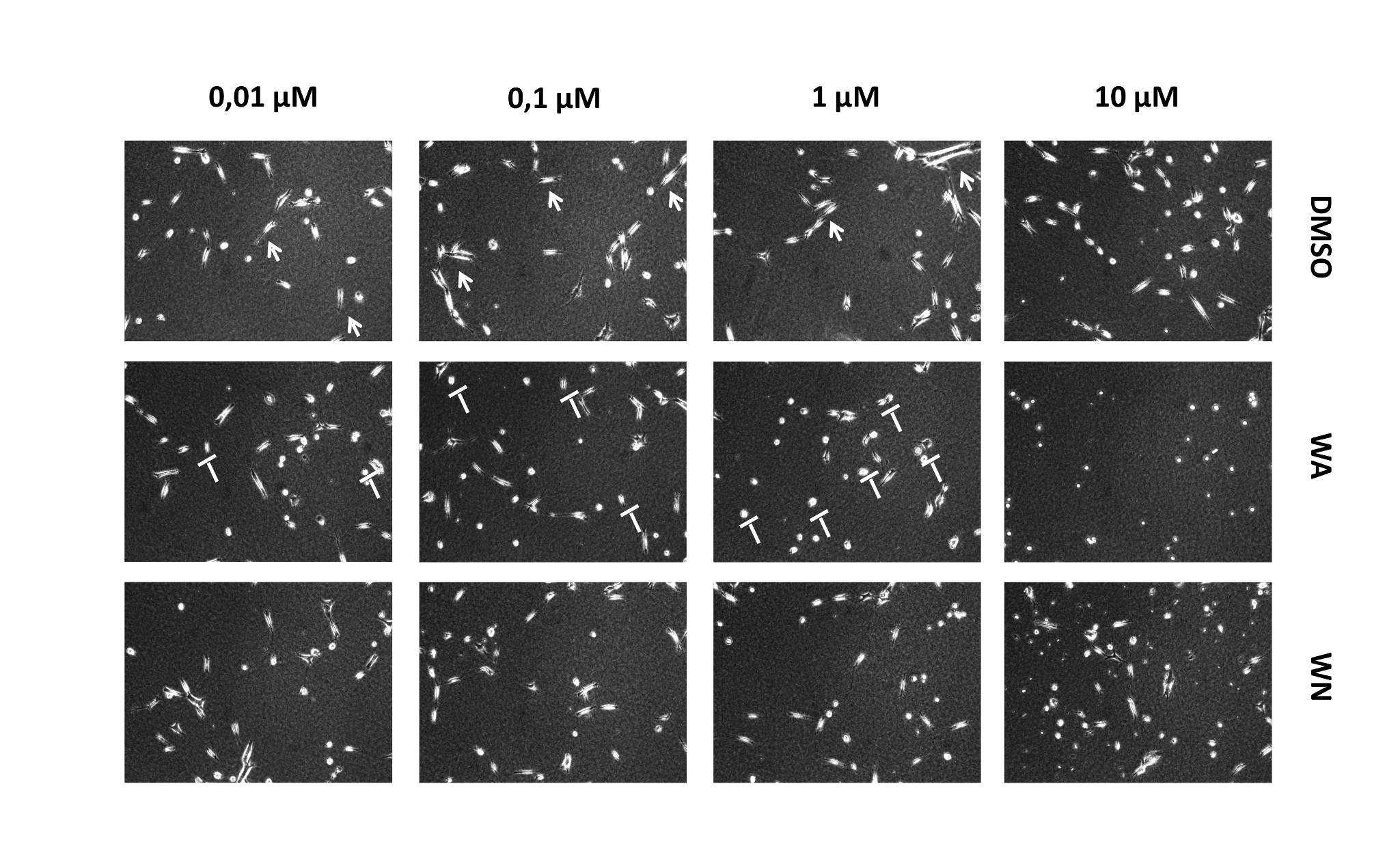

Supplement: Figure S2 — Representative phase-contrast microscopy pictures depicting decreased MDA-MB-231 cell invasion following WA, but not WN, treatment as determined by a 24-hour collagen type-I invasion assay. The quantity of solvent in ‘Solv’ was matched with the amount of DMSO in the corresponding treatment. Invasive cells are indicated with white arrows; non-invasive cells are indicated in the middle panel. (TIF) [file pone.0087850.s002.tif]

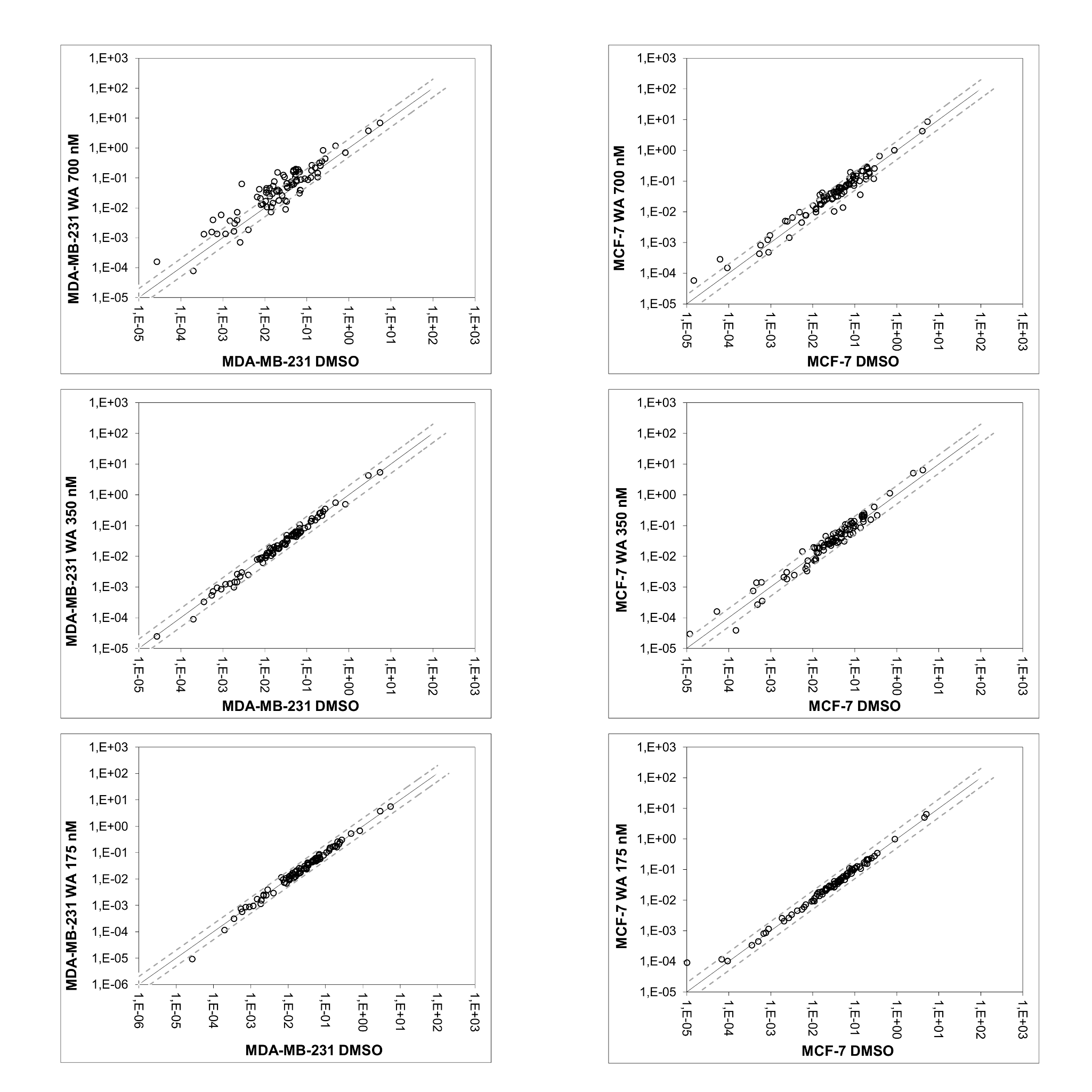

Supplement: Figure S3 — Concentration-dependent regulation of chromatin modifying enzyme gene expression by WA in MDA-MB-231 and MCF-7 cells. The scatter plots of the mean 2−ΔCt normalized expression values of each gene in the control samples (MDA-MB-231 DMSO, MCF-7 DMSO) versus the test samples (set-ups with WA) reveal the largest epigenetic plasticity of MDA-MB-231 cells exposed to the highest WA concentration (700 nM). The black line indicates fold change 2−ΔΔCt of 1. The dashed, gray lines indicate desired fold-change in gene expression threshold, here defined as 2. (TIF) [file pone.0087850.s003.tif]
