# Supplementary material for: Pharmacological Levels of Withaferin A (Withania somnifera) Trigger Clinically Relevant Anticancer Effects Specific to Triple Negative Breast Cancer Cells
Source: PLoS One. 2014 Feb 3;9(2):e87850. doi: 10.1371/journal.pone.0087850 (PMC3912072; doi:10.1371/journal.pone.0087850)
Supplement: Table S1 — A list of experimentally validated ChIP- sequencing data for genes regulated by E2F1 transcription factor in MCF-7 breast cancer cells. (DOCX) [file pone.0087850.s004.docx]

| **List of promoters positive for: HA-E2F1** | | | | | |
| --- | --- | --- | --- | --- | --- |
| **Cell line: MCF-7** | | | | | |
| **Genome: hg19** | | | | | |
| **ID** | **GENE** | **CHR** | **PROM_START** | **PROM_END** | **STRAND** |
| NM_000389 | CDKN1A | chr6 | 36646005 | 36646505 | + |
| NM_001012662 | SLC3A2 | chr11 | 62623033 | 62623533 | + |
| NM_001012664 | SLC3A2 | chr11 | 62623033 | 62623533 | + |
| NM_001013251 | SLC3A2 | chr11 | 62647893 | 62648393 | + |
| NM_001024628 | NRP1 | chr10 | 33623783 | 33624283 | - |
| NM_001024629 | NRP1 | chr10 | 33623783 | 33624283 | - |
| NM_001025248 | DUT | chr15 | 48623170 | 48623670 | + |
| NM_001025249 | DUT | chr15 | 48623170 | 48623670 | + |
| NM_001029989 | KIAA0101 | chr15 | 64673652 | 64674152 | - |
| NM_001067 | TOP2A | chr17 | 38574152 | 38574652 | - |
| NM_001071 | TYMS | chr18 | 657153 | 657653 | + |
| NM_001135733 | TP53INP1 | chr8 | 95961565 | 95962065 | - |
| NM_001178010 | CDC45 | chr22 | 19466963 | 19467463 | + |
| NM_001178011 | CDC45 | chr22 | 19466963 | 19467463 | + |
| NM_001197320 | MCL1 | chr1 | 150552164 | 150552664 | - |
| NM_001220778 | CDKN1A | chr6 | 36646036 | 36646536 | + |
| NM_001244972 | NRP1 | chr10 | 33623783 | 33624283 | - |
| NM_001244973 | NRP1 | chr10 | 33623783 | 33624283 | - |
| NM_001258315 | ECT2 | chr3 | 172468024 | 172468524 | + |
| NM_001258316 | ECT2 | chr3 | 172468024 | 172468524 | + |
| NM_001270472 | MCM3 | chr6 | 52149629 | 52150129 | - |
| NM_001786 | CDK1 | chr10 | 62537761 | 62538261 | + |
| NM_001798 | CDK2 | chr12 | 56360105 | 56360605 | + |
| NM_001948 | DUT | chr15 | 48623914 | 48624414 | + |
| NM_002388 | MCM3 | chr6 | 52149629 | 52150129 | - |
| NM_002394 | SLC3A2 | chr11 | 62623033 | 62623533 | + |
| NM_002689 | POLA2 | chr11 | 65028981 | 65029481 | + |
| NM_003362 | UNG | chr12 | 109535472 | 109535972 | + |
| NM_003504 | CDC45 | chr22 | 19466965 | 19467465 | + |
| NM_003538 | HIST1H4A | chr6 | 26021456 | 26021956 | + |
| NM_003539 | HIST1H4D | chr6 | 26189254 | 26189754 | - |
| NM_003540 | HIST1H4F | chr6 | 26240203 | 26240703 | + |
| NM_003541 | HIST1H4K | chr6 | 27799255 | 27799755 | - |
| NM_003542 | HIST1H4C | chr6 | 26103725 | 26104225 | + |
| NM_003543 | HIST1H4H | chr6 | 26285677 | 26286177 | - |
| NM_003544 | HIST1H4B | chr6 | 26027430 | 26027930 | - |
| NM_003545 | HIST1H4E | chr6 | 26204422 | 26204922 | + |
| NM_003873 | NRP1 | chr10 | 33623783 | 33624283 | - |
| NM_004091 | E2F2 | chr1 | 23857662 | 23858162 | - |
| NM_004417 | DUSP1 | chr5 | 172198153 | 172198653 | - |
| NM_005915 | MCM6 | chr2 | 136633997 | 136634497 | - |
| NM_005956 | MTHFD1 | chr14 | 64854308 | 64854808 | + |
| NM_006739 | MCM5 | chr22 | 35795665 | 35796165 | + |
| NM_014736 | KIAA0101 | chr15 | 64673652 | 64674152 | - |
| NM_016937 | POLA1 | chrX | 24711613 | 24712113 | + |
| NM_018098 | ECT2 | chr3 | 172468024 | 172468524 | + |
| NM_018518 | MCM10 | chr10 | 13203103 | 13203603 | + |
| NM_021960 | MCL1 | chr1 | 150552164 | 150552664 | - |
| NM_021968 | HIST1H4J | chr6 | 27791452 | 27791952 | + |
| NM_033285 | TP53INP1 | chr8 | 95961565 | 95962065 | - |
| NM_033379 | CDK1 | chr10 | 62537761 | 62538261 | + |
| NM_052827 | CDK2 | chr12 | 56360105 | 56360605 | + |
| NM_080911 | UNG | chr12 | 109534948 | 109535448 | + |
| NM_182751 | MCM10 | chr10 | 13203103 | 13203603 | + |
| NM_182763 | MCL1 | chr1 | 150552164 | 150552664 | - |
