# Supplementary material for: Pharmacological Levels of Withaferin A (Withania somnifera) Trigger Clinically Relevant Anticancer Effects Specific to Triple Negative Breast Cancer Cells
Source: PLoS One. 2014 Feb 3;9(2):e87850. doi: 10.1371/journal.pone.0087850 (PMC3912072; doi:10.1371/journal.pone.0087850)
Supplement: Table S2 — A list of experimentally validated ChIP- sequencing data for genes regulated by H3K4me2,me3 in Human Mammary Epithelial Cells (HMEC). (DOCX) [file pone.0087850.s005.docx]

| **List of promoters positive for: H3K4me2,me3** | | | | | |
| --- | --- | --- | --- | --- | --- |
| **Cell line: HMEC** | | | | | |
| **Genome: hg19** | | | | | |
| **ID** | **GENE** | **CHR** | **PROM_START** | **PROM_END** | **STRAND** |
| NM_001067 | TOP2A | chr17 | 38574152 | 38574652 | - |
| NM_001195053 | DDIT3 | chr12 | 57914250 | 57914750 | - |
| NM_001195056 | DDIT3 | chr12 | 57914250 | 57914750 | - |
| NM_001195057 | DDIT3 | chr12 | 57914250 | 57914750 | - |
| NM_001199741 | GADD45A | chr1 | 68150409 | 68150909 | + |
| NM_001258315 | ECT2 | chr3 | 172468024 | 172468524 | + |
| NM_001258316 | ECT2 | chr3 | 172468024 | 172468524 | + |
| NM_001270472 | MCM3 | chr6 | 52149629 | 52150129 | - |
| NM_001786 | CDK1 | chr10 | 62537761 | 62538261 | + |
| NM_001924 | GADD45A | chr1 | 68150409 | 68150909 | + |
| NM_002388 | MCM3 | chr6 | 52149629 | 52150129 | - |
| NM_002970 | SAT1 | chrX | 23800824 | 23801324 | + |
| NM_004083 | DDIT3 | chr12 | 57914250 | 57914750 | - |
| NM_005949 | MT1F | chr16 | 56691404 | 56691904 | + |
| NM_005952 | MT1X | chr16 | 56715931 | 56716431 | + |
| NM_018098 | ECT2 | chr3 | 172468024 | 172468524 | + |
| NM_018492 | PBK | chr8 | 27695299 | 27695799 | - |
| NM_033379 | CDK1 | chr10 | 62537761 | 62538261 | + |
